# Brain death due to intracranial hemorrhage in a child following suspected *Bothrops* snakebite

**DOI:** 10.1590/0037-8682-0264-2024

**Published:** 2024-12-16

**Authors:** Heloisa Dellandrea, Camilo Molino Guidoni, Arnildo Linck, Edmarlon Girotto

**Affiliations:** 1 Universidade Estadual de Londrina, Centro de Ciências da Saúde, Curso de Medicina, Londrina, PR, Brasil.; 2 Universidade Estadual de Londrina, Departamento de Ciências Farmacêuticas, Londrina, PR, Brasil.; 3 Universidade Estadual de Londrina, Departamento de Pediatria e Cirurgia Pediátrica, Londrina, PR, Brasil.

**Keywords:** *Bothrops* genus, Cerebral hemorrhage, Brain death

## Abstract

Snakebites from the genus *Bothrops* are common and are responsible for the highest mortality rate in Brazil. Factors related to the species, treatment, and patient influence the clinical manifestations and prognosis of the condition. Young patients without comorbidities have better prognoses and rarely develop severe systemic complications. This report discusses a case of brain death of an 11-year-old boy due to intracranial hypertension following possible *Bothrops* snakebite. Despite receiving antivenom therapy, the patient experienced seizures, mental confusion, and decreased consciousness. Autopsy, epidemiology, clinical presentation, and laboratory results indicated a snakebite with unconventional symptoms as the cause of death.

## INTRODUCTION

 Toxins produced by the most prevalent **pit vipers** in Brazil typically increase vascular permeability and leukocyte infiltration. This, combined with other actions of the venom components, leads to edema, blistering, local hemorrhage, and pain, sometimes progressing to ischemia, necrosis, and systemic complications. The triggering of inflammatory responses and coagulation disorders, such as defibrination and thrombocytopenia, may cause endothelial damage to the cerebral capillaries, resulting in intracerebral hemorrhages^1^ ^,^ ^2^. The composition of venom is influenced by habitat, sex, diet, or ontogenetic development. Young animals of the same species may produce more anticoagulant toxins, whereas adults exhibit predominantly inflammatory and proteolytic actions^3^ ^,^ ^4^. The venom of some species is known for a higher mortality rate^5^.

Despite the low mortality rate, some factors that negatively affect prognosis include existing cardiovascular diseases^6^, time to antivenom administration, bite location, immunity, and the amount of venom injected^7^. Cerebral hemorrhagic events following antivenom therapy are underreported, particularly in pediatric patients without comorbidities. Reviews in the Cochrane Library, LILACS, SciELO, MEDLINE, PubMed, and PubMed Central found a single case study from 2013 that addressed cerebral hemorrhage after a *Bothrops* snakebite in a child^2^. However, delayed management of more than 24 hours was reported.

## CASE REPORT

**FIGURE 1:**
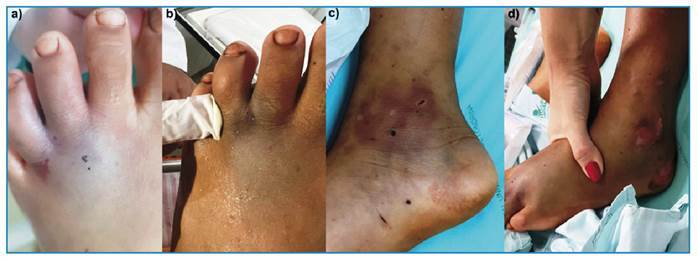
Photos taken at the University Hospital of Londrina showing the area of the possible bite. **(a, b)** Two punctate lesions and hemorrhage points can be observed in the distal region of the third left metatarsal. **(c, d)** Multiple lesions of unknown etiology on the left foot.

An 11-year-old male with a snakebite on the left ankle, 50 minutes earlier, was admitted to a small hospital. The patient presented with gingival bleeding, bruising, agitation, and confusion. According to his mother, they were walking in a pasture in a rural area when he felt a sudden, sharp pain, similar to being pricked by a thorn. The mother reported seeing a snake at the site, but it fled before being identified. A few minutes later, the patient experienced a seizure and was taken to a healthcare facility. Examination revealed bruised areas with punctate lesions on the distal third metatarsal and left medial malleolus, indicative of bite marks. Additionally, there was mild, non-indurated, and non-progressive edema of the distal third metatarsal and other unidentified lesions (Figure 1).

The patient was referred to a larger hospital and the Poison Information and Assistance Center (CIATox) was contacted for an evaluation. Suspecting a snakebite, the CIATox recommended administration of 12 vials of *Bothrops* antivenom, which commenced approximately two hours after the incident. 

**FIGURE 2:**
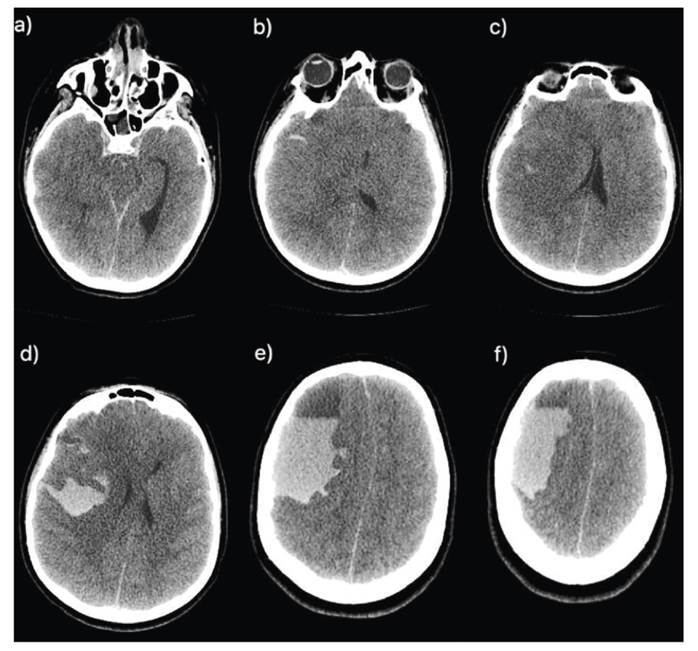
Sequence of right frontal hypoattenuating images, with a collection of hematic content, indicating a compressive effect on the adjacent brain parenchyma, characterized by erasure of the sulci and fissures, compression on the lateral ventricles and III ventricle, and trans tentorial herniation, in addition to a midline shift to the left **(a, b, c)**. Intraparenchymal hematoma, with hematic content permeating grooves and fissures, compatible with subarachnoid hemorrhage. Effacement of grooves and fissures suggesting diffuse cerebral edema **(d, e, f)**.

Initially, the patient showed improvement in the agitation and mental confusion. However, a few hours after antivenom therapy, his condition worsened significantly, necessitating referral to a tertiary healthcare facility. Upon arrival, the patient developed shock, refractory to catecholamines, requiring vasopressin and invasive ventilatory support. For cerebral edema, hypertonic saline, anticonvulsant prophylaxis and analgosedation were administered. Computed tomography (CT) revealed active hemorrhage, possible anasarca, and a large cerebral hemorrhagic area (Figure 2). 

Blood collected 11 hours post-bite showed abnormalities in: partial thromboplastin time (KPTT) 42.3s (24-30 seconds), prothrombin time 23.4s (<15s), prothrombin activity 39.1% (70-110%), International Normalized Ratio 1.96 (0.8-1.2), fibrinogen 33 mg/dL (150-450 mg/dL), along with anemia and leukocytosis. Approximately 18 hours after the snakebite, in a critical state and without sedation, the patient had fixed mydriatic pupils and limb areflexia. Follow-up 21 hours post-bite showed normalization of coagulation and improvement in the blood count. However, hematuria and an increased high-sensitivity troponin level of 910 ng/L (<60.4 ng/L), creatine phosphokinase (CK) 1311 U/L (39-308 U/L), and CK-myocardial band 12.2 ng/mL (<3.6 ng/mL) were noted.

Due to unresponsive and areflex coma and suspicion of intracranial hypertension on optic nerve ultrasonography performed by the neurosurgery team, later confirmed by head CT, brain death was suspected and confirmed 50 hours after admission using a protocol recommended by the Ministry of Health. During autopsy, external physical examination revealed two puncture wounds approximately three centimeters apart on the medial malleolus, surrounded by a purplish bruise, and multiple punctate lesions, without bruises, on the right foot and ankle. The forensic pathologist determined the cause of death to be cerebral hemorrhage due to a snakebite.

## DISCUSSION

Despite the high frequency of clinical manifestations, the incidence of fatal complications from *Bothrops* spp. bites is low and rarely reported in the literature^8^. Venom components, mainly zinc-dependent metalloproteases, phospholipase A2, and serine proteases initiate significant inflammatory responses. In addition to localized direct tissue damage caused by toxins, these components trigger complement system activation and leukocyte recognition^9^. The predominant systemic effects include coagulopathy or incoagulability caused by platelet inhibition or activation by toxins^3^ ^,^ ^4^ ^,^ ^9^. Intracerebral hemorrhage is associated with thrombocytopenia and severe incoagulability as well as endothelial damage to cerebral capillaries by the hemorrhage and the toxins individually^2^.

The venom composition varies among species and is influenced by snake age. Although the impact of this variation on clinical outcomes is well-known, it is not fully understood. Species such as *B. caribbaeus*, *B. asper* and *B. lanceolatus* produce toxins that reduce the prothrombin level and increase the fibrinogen level, thus exhibiting procoagulant potential. Conversely, *B. jararaca* and *B. atrox* produce fibrinolytic substances that degrade fibrin or lower coagulation factors, thereby predisposing to systemic hemorrhage^9^. 

De Roodt *et al* ^10^ reported that in*B. alternatus*, the distance between the fangs and puncture marks can indicate the body length of the snake. It was also verified that the amount of venom injected is proportional to the size of the animal and to the amount of antivenom required for treatment. In the present report, the distance between the punctures observed during the autopsy, accounting for the variation caused by edema, could indicate the fangs of a larger animal, ranging from 75 cm to over 100 cm in length^10^.

Regarding the prognosis, more rapid initiation of antivenom therapy improves the likelihood of recovery. However, even with early treatment, some patients develop fatal complications^7^. In cases where the snake is not identified, medical management should be based on clinical manifestations, epidemiological relationships should be considered, and differential diagnoses should be pursued to address the possibility of other conditions. 

In this case report, the patient presented with local hemorrhage, history of gingival hemorrhage, bruising, and mild edema. Less specific symptoms, such as confusion, agitation, and decreased consciousness have also been observed in case reports of hemorrhagic complications typically associated with central nervous system involvement^5^ ^,^ ^8^ ^,^ ^11^ ^-^ ^13^.

Although early convulsive episodes are atypical in snakebites, several mechanisms have been reported as possible causes. First, the convulsions may have been induced by the snake venom by itself. Second, the occurrence of multiple rapidly developing cortical infarcts resulting from generalized endothelial injury, may have been induced by the venom^14^. This aligns with the patient experiencing a significant intracranial hemorrhage.

Throughout treatment, the vital signs of the patient fluctuated, with hypotension and tachycardia, but a normal temperature and respiratory rate. Initial biochemical analysis revealed anemia, increased segmented cell and leukocyte counts, and coagulation abnormalities. Several hours later, mild hematuria was observed, and the coagulation test results normalized. However, at that time, the frontal hemorrhage and increased intracranial pressure progressed to an irreversible state.

One hypothesis explaining the clinical manifestations is that the venom of a young *Bothrops* snake may have a stronger anticoagulant action and a higher likelihood of causing hemorrhagic events^3^ ^,^ ^4^. Silveira *et al.* (2016)^12^ and Pinho (2001)^5^ described cases involving young *Bothrops* snake bites in adults, in which the bites led to predominantly hemorrhagic manifestations with less proteolytic activity^3^ ^,^ ^4^ ^,^ ^5^ ^,^ ^12^. In both instances the patients exhibited similar severe symptoms, including anemia, prolonged coagulation time, loss of consciousness, and disorientation. The first patient experienced signs of intracerebral hemorrhage, while the second patient, bitten by *B. jararacussu*, despite receiving antivenom therapy, progressed to a comatose state. This patient also presented with anisocoria, neck stiffness, hemoglobinuria, diffuse retinal hemorrhage, and cardiorespiratory arrest 28 hours later.

Although cardiovascular and coagulopathy comorbidities increase the risk of severe complications, they can also lead to fatality in patients without any reported comorbidities. Oliveira (2017)^6^ and Delgado (2017)^11^ described two adult patients with no previous comorbidities who had been bitten by *Bothrops* snakes. Both patients received the appropriate antivenom therapy. The first patient had tachycardia, sialorrhea, aphasia, mydriatic non-reactive pupils, and hypotension, and presented with intraparenchymal and subarachnoid hemorrhage with intracranial hypertension as the cause of death^6^. The latter manifested as hypertension and left hemiparesis, and despite right ventricular system hemorrhage, he showed improvement in motor and speech functions on the following days^11^.

Battelino *et al.* (2003)^13^ evaluated the efficacy of *B. jararaca* antivenom on microcirculatory effects in rats. This showed that administering antivenom 15 minutes before, simultaneously, or 15 minutes after venom inoculation did not completely reverse the effects of the venom. In all cases, the rats exhibited some degree of coagulation disorder, possibly due to delayed interaction between the venom and antivenom within the blood vessels. This highlights the possibility that patients may develop irreversible complications before benefitting from antivenom therapy^13^.

Pediatric patients usually have a good prognosis; however, fatal complications in young individuals without comorbidities are not unprecedented. Pardal *et al.* (2015)^2^ reported the case of a 10-year-old child who was bitten on the foot and initially presented with local pain and edema. Owing to logistical difficulties, he arrived at the medical facility one day later with extensive edema in the affected limb, bruising, blisters, and hematuria, and entered a comatose state. Within two days, the patient received a total of twelve vials of *Bothrops* antivenom. Three days later, the patient developed right hemiplegia, left-sided lip deviation and signs of local infection. A CT scan showed hemorrhagic brain lesions, and laboratory tests showed, red blood cell count 3.61 million/mm³, hemoglobin 10.3 g/dL, hematocrit 31.1%, 90% segmented cells, KPTT 20.3s, leukocytes 6.54/mm³, and CK 915 IU/mL^2^, suggesting anemia and acute inflammation, which align with the findings in the present case study.

Although the snake was not identified in the current case, clinical, laboratory, and epidemiological evidence strongly suggests the presence of a *Bothrops* snakebite. The child had no previously reported comorbidities, was admitted to the healthcare facility within one hour of clinical presentation, and promptly received antivenom therapy. Despite all preventive measures, clinical manifestations have multifactorial origins, and there are still poorly understood factors related to venom and antivenom therapy that may have influenced the development of sudden intracranial hemorrhage that could not be treated in a timely manner. Thus, healthcare services and professionals must focus on specialized and prompt care through consultation with the CIATox, investigation of undiagnosed comorbidities, and pay close attention to symptoms that differ from typical manifestations. This approach is essential to mitigate complications and prevent fatalities within the scope of clinical management.

## ETHICS

This case report was approved by the Institutional Ethics Committee, CAAE 80320024.0.0000.5231, under the approval number 6.986.288/2024.
